# Susceptibility‐induced distortion correction in hyperpolarized echo planar imaging

**DOI:** 10.1002/mrm.26839

**Published:** 2017-07-19

**Authors:** Jack J. Miller, Angus Z. Lau, Damian J. Tyler

**Affiliations:** ^1^ Department of Physiology Anatomy & Genetics, Sherrington Building, University of Oxford Oxford United Kingdom; ^2^ Department of Physics Clarendon Laboratory, University of Oxford Oxford United Kingdom; ^3^ Health Sciences, Sunnybrook Research Institute Toronto Ontario Canada

**Keywords:** magnetic resonance spectroscopy, hyperpolarized 13 C, echo planar imaging, distortion correction

## Abstract

**Purpose:**

Echo planar imaging is an attractive rapid imaging readout that can image hyperpolarized compounds in vivo. By alternating the sign of the phase encoding gradient waveform, spatial offsets arising from uncertain frequency shifts can be determined. We show here that blip‐reversed echo planar imaging can also be used to correct for susceptibility and *B*_0_ inhomogeneity effects that would otherwise produce image‐domain distortion in the heart.

**Methods:**

Previously acquired blip‐reversed cardiac 3D‐Spectral‐Spatial echo planar imaging volumetric timecourses of hyperpolarized [1‐^13^C]pyruvate were distortion corrected by a deformation field estimated by reconstructing signal‐to‐noise ratio (SNR)‐weighted progressively subsampled temporally summed images of each metabolite.

**Results:**

Reconstructing blip‐reversed data as proposed produced volumetric timecourses that overlaid with proton reference images more consistently than without such corrections.

**Conclusion:**

The method proposed may form an attractive method to correct for image‐domain distortions in hyperpolarized echo planar imaging experiments. Magn Reson Med 79:2135–2141, 2018. © 2017 The Authors Magnetic Resonance in Medicine published by Wiley Periodicals, Inc. on behalf of International Society for Magnetic Resonance in Medicine. This is an open access article under the terms of the Creative Commons Attribution License, which permits use, distribution and reproduction in any medium, provided the original work is properly cited.

## INTRODUCTION

Hyperpolarized [1‐^13^C]pyruvate forms a versatile metabolic probe that has been used extensively to quantify cardiac metabolism and pH in health and disease 1, 2, 3, 4, 5. Many conditions where metabolic dysregulation is implicated are often spatially localized, such as myocardial ischaemia. It is challenging to image hyperpolarized compounds in the heart for numerous reasons, predominantly 1 cardiac motion, which cannot readily be retrospectively corrected for as the observed hyperpolarized signals are inherently a function of time; and 2 the finite, decaying, and nonrenewable magnetization “reservoir” generated by hyperpolarization methods necessitates magnetization‐efficient rapid imaging techniques. These issues have lead to a variety of strategies to image metabolism following the injection of hyperpolarized [1‐^13^C]pyruvate, such as EPSI 6, IDEAL CSI 7, multiband excitation based approaches 8, and spectral‐spatial excitation with a variety of rapid imaging trajectories, including echo planar imaging (EPI) 9, 10, 11, 12, 13, 14, 15. Many of these methods use EPI in part because it is one of the oldest proposed rapid imaging strategies 16, 17, 18, is fast, commonly available on commercial scanners, and produces artefacts that are analytically understood.

EPI artefacts fall broadly into two categories: artefacts arising from gradient infidelity, such as the the Nyquist $\left(\frac{N}{2}\right)$ ghost, caused by eddy currents predominantly imposing an alternating phase error between different lines of k‐space 19; and image‐domain distortions such as compressions, expansions, tearings, and other more complex geometrical transformations that arise from susceptibility changes and their associated *B*_0_ effects 20. In proton EPI, where image domain signal‐to‐noise ratio (SNR) is high, a large number of methods have been proposed to ameliorate or eliminate these imperfections, such as the use of navigator echoes to remove the linear Nyquist ghost 21, and the direct measurement of *B*_0_ to correct for geometric distortions through the use of reference scans 22, 23.

Additionally, the comparatively small bandwidths used in EPI acquisitions mean that the acquired images are prone to linear displacements if the transmitter frequency and that of the chemical species imaged differ. By alternating the sign of the phase encoding gradient between successive EPI acquisitions, Cunningham et al. have shown that it is possible to correct for the inherent uncertainty in transmitter frequency with in vivo hyperpolarized experiments by shifting both images until they overlap with each other, either manually or through the use of information theory metrics such as mutual information 24. Recently, acquisition‐based methods have been proposed to ameliorate these distortions in by acquiring dual‐echo EPI data, and estimating field shifts directly at the expense of increased echo time (TE) 25. In proton spin‐echo diffusion weighted imaging (DWI)‐EPI images, Andersson et al. 26 have likewise analytically shown how *B*_0_ and susceptibility effects result in geometric distortions, and provide an image domain formalism through which the generalized distortion field **K** can be determined given the k‐space trajectory, knowledge of *B*_0_ and other sequence parameters such as the acquisition bandwidth.

## METHODS

### Theory

As described previously 23, 26, it is helpful to consider the EPI experiment in a linear algebra framework. If a slice of magnetization has been excited, then it is well‐known that, neglecting relaxation, the signal received S(t) is

(1) $$S(t)\propto \int \int (x,\;y)e^{iy\left(\Delta B_{0}(x,\;y)+G_{f}(x,\;y,\;t)+G_{p}(x,\;y,\;t)\right)}\;dxdy,\text{or}\;\text{equivalently},\;s=A\rho$$

in a matrix form, where *G_f_* and *G_p_* represent the time‐integrals of the frequency‐encoding and phase‐encoding waveforms respectively. If $\Delta B_{0}=0$ everywhere, then **A** is a 2D Fourier matrix; let us denote this special case **F**. If this is not the case then one can define a mapping between the “true” EPI image ρ, and **f** the acquired, distorted data, as **f** = **F** ^†^ $A\rho \overset{\text{def}}{=}K\rho$. If **K** could be estimated (given knowledge of the proposed k‐space trajectory), then this implies that undistorted images could be estimated from the acquired data as $\hat{\rho }=K^{-1}f$.

Unfortunately, as **K** ultimately can represent a many‐to‐one or many‐to‐many mapping between sets of images, its inverse does not necessarily exist, and its Moore–Penrose pseudo inverse is not guaranteed to be numerically constructible: image information may be obscured by artefacts. However, as quantitatively shown in detail elsewhere 26, by acquiring blip‐reversed pairs of EPI images it is possible to acquire datasets with two distinct deformation fields, $K^{+}$ and $K^{-}$, and obtain a preconditioned numerical problem that is computationally tractable. If knowledge of *B*_0_ is not otherwise present, it can be shown that the deformation field $b\in {\mathbb{R}}^{3}$ that is consistent with the observed data is that for which

(2) $$\underset{{}_{b}}{\arg\min}\left(\sum_{c=1}^{m}\;\left[\;f_{c+}^{⊺}\;f_{c-}^{⊺}\right]\;\;R_{c}(b)\;\left[\begin{array}{c} f_{c+}\\ f_{c-} \end{array}\right]\right)$$

where the sum runs over all phase encoding columns *c* and **R** is a distortion operator defined in terms of $K^{\pm }(b)$. This problem is usually directly solved in a least‐squares sense with parameters and starting values optimized for human neuroimaging by the program “topup” in the FMRIB software library (FSL) suite 27.

In the context of imaging hyperpolarized [1‐^13^C]pyruvate metabolism, it is not immediately clear how to construct **K** ^±^ given that the hyperpolarized EPI experiment obtains a stack of 3D images of 1… M different metabolites acquired at time points 1…T. Each image $f_{i}$ therefore varies in both intensity and SNR over time. We expect that *B*_0_ shifts between different metabolites are common between them, whereas gross translations will occur along the readout axis arising from the uncertainty in transmitter/receiver frequency set prior to the start of the hyperpolarized experiment relative to that of the metabolite in question.

As a consequence, it is necessary to create a pair of **f** ^±^ images containing a region of sufficient information to provide a compact support for the algorithm defined by Equation (2) to adequately function. This is tantamount to ensuring that the condition number of **R** is reasonable, and that information encompassing all metabolites is acquired. The returned deformation fields can then be applied to all metabolites, producing two sets of undistorted images for each metabolite. Any residual alternating spatial shifts between the two unwarped images can be corrected at this point by an affine coregistration step, producing two mappings, **a** which maps $\rho^{+}$ onto $\rho^{-}$, say, and **b**, which maps $\rho^{-}$ onto $\rho^{+}$. In the absence of experimental noise and numerical errors, $a=b^{-1}$. We require a mapping to the same frame as the proton reference, and in the presence of noise we propose the geometric mean construct $\sqrt{\sqrt{b}\sqrt{a}}$ to as an interpolator to the proton image space, where $\sqrt{a}$ denotes the matrix square root of **a** whose eigenvalues lie in the upper half plane.

We present the following algorithm to perform these tasks:

1. Reconstruct EPI data acquired, correcting for the Nyquist ghost and performing a multicoil recombination if appropriate.
2. For each metabolite, correct for bulk frequency shifts by grossly aligning the blip‐up and blip‐down images along the readout direction, either manually or through iteration via an information‐theory metric, such as mutual information or image self‐similarity. Partial voxel shifts are implemented by applying a phase ramp in the Fourier domain.
3. Construct a pair of blip‐up/blip‐down SNR‐weighted mean images $f_{\pm }$ from all metabolites acquired, scaled to maximum metabolite level. We performed this via obtaining a mean of the SNR‐weighted sum of each metabolite time course, i.e., if *σ_tm_* represents the relative noise (relative to maximum signal) term in image $X_{xyztm}^{\pm }$ where *t* represents time, *m* metabolite, and the ± label denotes the overall sign of the phase encoding gradient waveform, compute
  (3) $$f^{\pm }=\frac{1}{M\sum_{tm}\left(1/\sigma_{tm}^{2}\right)}\sum_{m=1}^{M}\sum_{t=1}^{T}\frac{1}{\sigma_{tm}^{2}}X_{xyztm}^{\pm }.$$
4. Obtain $b^{\mp }$ by applying topup to $f^{\pm }$, here with three levels of progressive subsampling (by factors of 4, 2, and 1) with corresponding Gaussian blurring (FWHM 8, 4, 0 pixels, respectively). A large regularization parameter was found to be required to ensure that the deformation field is smooth and slowly varying, and not unduly informed by noise and the typical disconnected field of view (FOV) that hyperpolarized datasets have compared to the brain ( λ=50, 20, 2).
5. Apply the computed $b^{\mp }$ to each acquired image *X* to estimate a stack of undistorted images.
6. Correct for residual frequency shifts (i.e., alternating translations) via the affine registration algorithm separately for each metabolite, and obtain two (Hermitian) matrices c,d mapping between the sets of images.
7. Construct the interpolator $e=\sqrt{\sqrt{c}\sqrt{d}}$ (here via a Schur method 28, 29), apply it, and hence obtain a distortion‐corrected registered volumetric stack for each time point and metabolite. Note that the Hermiticity of c, d ensure that their square root exists.

The above algorithm was implemented in MATLAB and does not present a significant computational workload, requiring a few seconds to operate on 64×64×32×20×3 matrix sizes on a Core i7–3370k workstation computer (running Linux 4.4.49‐generic) with 16 GiB RAM and an NVidia Titan X GPU. Its source code (together with the experimental datasets described subsequently) can be freely downloaded from http://github.com/NeutralKaon/HPTopup.

### Experimental Methods

As described previously 12, data were acquired using a 3D spectral‐spatial EPI sequence with flyback excitation and a centric‐ordered k‐space trajectory that imaged [1‐^13^C]pyruvate, ^13^C‐bicarbonate, and [1‐^13^C]lactate in the myocardium following the intravenous infusion of [1‐^13^C]pyruvate into three anaesthetized healthy rats. Approximately 2 mL of prepolarized pyruvate was manually infused over 20 s via a tail vein cannula. The acquisition matrix size was 32×16×12, FOV $64\times 32\times 48{\;\text{mm}}^{3}$, TE =16.34 ms, sequence length =31.48 ms, with the flip angle θ= 3° for pyruvate, or θ= 20° otherwise. As each shot was prospectively gated, the repetition time (TR) was 1 RR interval, ≈150 ms. The order of acquisition was $\left[\ldots ,\;{\text{Pyr}}^{+},\;{\text{Bic}}^{+},\;{\text{Lac}}^{+},\;{\text{Pyr}}^{-},\;{\text{Bic}}^{-},\;{\text{Lac}}^{-},\;\ldots \right]$ where ${}^{\pm }$ denotes the sign of the phase encoding gradient waveform. A two channel volume transmit/surface receive coil was used, with multicoil data recombined by the method of McKenzie 30 (which phases each image to be almost entirely real) and additionally zero‐filled.

All animal experiments were performed according to relevant UK legislation. The data presented are reanalyzed versions of that acquired in the course of other ongoing studies, for which an explicit local ethical review procedure and independent cost/benefit analysis had been undertaken.

To investigate the improved spatial coregistration of the acquired hyperpolarized data with the technique, a simple phantom experiment was performed in which hyperpolarized pyruvate was injected into a syringe, and imaged as above. No shimming was performed in an attempt to mimic the variable *B*_0_ environment of the in vivo situation. As has been previously proposed to quantify EPI distortions 31, the Jaccard self‐similarity index was computed to compare the spatial distribution of acquired proton and carbon images pre‐ and post‐correction. The Jaccard index represents the area of intersection over the area of union of two binary sets *X_i_* and *X_j_*, and is defined as

(4) $$J_{ij}=\frac{\left|X_{i}\cap X_{j}\right|}{\left|X_{i}\cup X_{j}\right|},\;J_{ij}\in [0,\;1].$$

For the phantom data, a gradient echo proton reference image was obtained, and thresholded to provide a binary mask for comparison that indicated the location of the syringe body together with a urea phantom. The temporally summed hyperpolarized image was then thresholded, and the Jaccard index for this resulting mask compared with the proton “ground truth” pre‐ and post‐correction. Additionally, the odd‐ and even‐echo hyperpolarized images were temporally summed, thresholded, and compared with each other: assuming that the number of frames acquired is large compared with the dynamic process of injection, these two datasets should nominally be identical were it not for distortion.

Owing to the lack of an appropriate “ground truth” image for the acquired in vivo dataset, odd‐ and even‐echo data were temporally summed, thresholded, and the Jaccard index computed for these two sets, for each metabolite. In comparison to the phantom case, this process tacitly assumes that the frame‐rate (of 1.2 s/3D image) is high compared to any biological process that could conceivably lead to a difference between odd‐ and even‐echo images.

## RESULTS

**Figure 1:**
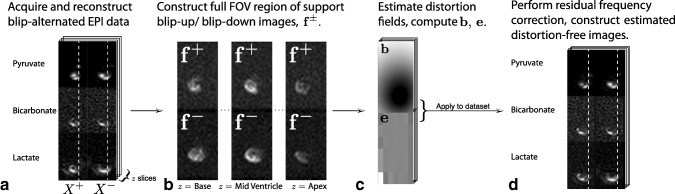
An overview of the proposed distortion correction algorithm, together with illustrative example images at each stage of the process. **a**: Hyperpolarized data was acquired with alternated phase encoding blip sign; shown here are representative single mid‐ventricular slice data from two adjacent time points with two different blip signs. The white dotted line illustrates nonuniform compressions and expansions arising from susceptibility effects near the heart. **b**: From these data, approximately equal‐SNR $f^{\pm }$ images are constructed over the whole imaging volume for distortion correction analysis. **c**: From **f** ^±^, compute $b^{\mp }$ and **e**. **d**: Hence construct estimated undistorted stacks of metabolic images, shown here at the same time points as in 1.

We found that the method proposed is able to correct for susceptibility artefacts arising in the rodent heart, even in the low SNR environment of hyperpolarized imaging experiments. A graphical summary of the method is shown in Figure 1, illustrating the nonlinear compression and expansion artefacts that can be present in EPI readouts (Fig. 1a), the subsequent calculation of the images **f** ^±^ (Fig. 1b), and distortion field **b** (Fig. 1c). This enables the subsequent amelioration of such artefacts (Fig. 1d).

**Figure 2:**
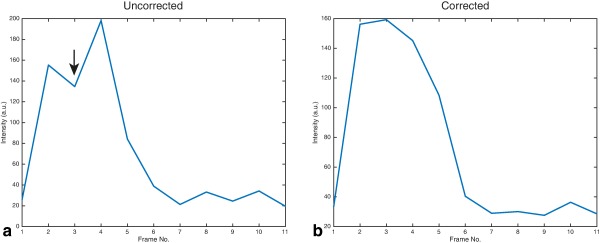
Intensity of a single voxel along the edge of the ventricular line drawn in Figure 1 through time, prior to (**a**) and after (**b**) the distortion correction algorithm. The increase in amplitude of the first marked frame (and the decrease in amplitude of the point immediately adjacent to it) is consistent with the removal of an expansion/compression artefact respectively, which would be expected to alternate between frames of the acquisition.

By plotting the through‐time behavior of a single voxel any compression/stretching artefacts present would be observed as alternating regions of high/low signal on alternate frames, as the object imaged “moves” between acquisitions. Figure 2 shows such a profile for a pyruvate voxel within the line profile shown in Figure 1, at the edge of the heart where susceptibility artefacts are expected to be greatest, both prior to and after the use of the proposed algorithm. Three example datasets processed with and without the proposed method are shown concatenated through space as Supporting Videos S1–S6. It is apparent that the alternating compression/extension artefact is ameliorated in these three distinct experiments.

**Figure 3:**
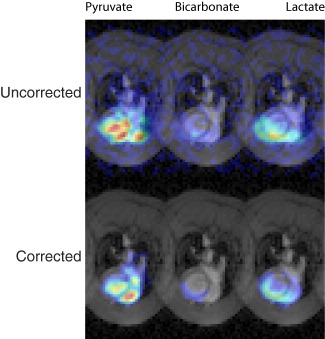
Basal single‐slice images of pyruvate, bicarbonate, and lactate summed over time and shown overlaid on a corresponding proton image. The color axis scale is common to both images, with transparent set to 0.2× the maximum signal obtained. As a consequence, the oscillating susceptibility errors are apparent in the summed image as a discrepancy between the observed anatomy and the expected signal location. The proposed method appears to ameliorate this, and shows pyruvate perfusion in the ventricles and metabolite production solely within the myocardium.

As illustrated in Figure 3, it was found that images reconstructed through the proposed technique had less apparent variation when overlaid onto simultaneously acquired proton references. Specifically, the oscillating compression/expansion distortions caused during the EPI readout combined with small frequency errors on the order of 20 Hz results in an apparent blurring in images when temporally summed, and hence potentially misleading discrepancies between metabolite location and anatomy. Ameliorating these distortions therefore reduced the apparent width in the reconstructed image domain, and additionally increased apparent SNR in the temporally summed images due to the “constructive” addition within voxels. Supporting Videos S7–S8 contain the contents of Figure 3 without the temporal summation, and illustrates the acquired distorted data (Supporting Video S7) and that after correction (Supporting Video S8).

**Figure 4:**
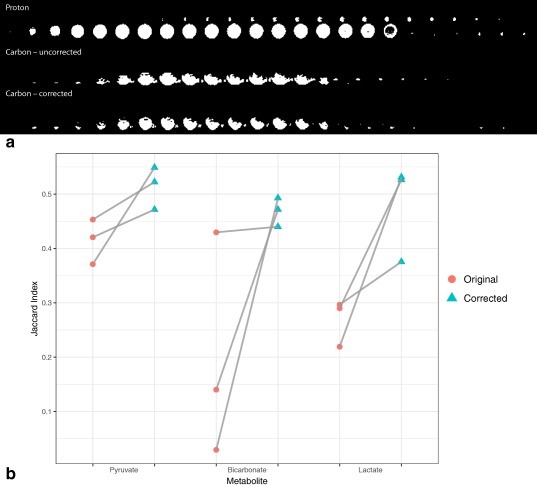
**a**: Proton (top), pre‐correction (middle), and post‐correction (bottom) thresholded masks obtained from the distortion correction process. While thresholding ameliorates the effects of the coil sensitivity profile to some degree, it is apparent that the reduced spatial extent of *B*_1_ in the carbon surface array used is still visible. **b**: The Jaccard index comparing temporally summed odd and even echoes before and after correction in vivo shows a significant increase in the similarity of the image masks (*P* < 0.05), reflecting reduced distortions.

For the case of the phantom scanned, it was found that the Jaccard index between the thresholded proton and carbon images was increased from 0.26 to 0.29 by the distortion correction method, with masks that are illustrated in Figure 4a. The effect of the differing coil sensitivity profiles in this comparison could not be easily corrected for. In contrast, the Jaccard index computed between temporally summed odd and even echoes — which should nominally be identical given the long *T*_1_ of pyruvate in water —increased from 0.29 to 0.76, reflecting the greatly ameliorated oscillating distortions in the images. We believe that this difference arises due to the smaller spatial extent of $B_{1}^{-}$ provided by the carbon surface receive array used.

In vivo, we found that the Jaccard index computed as described above was increased in every metabolite and every animal scanned, as shown in Figure 4b. The median score was significantly increased in every group when considered via a nonparametric ANOVA method (the Kruskal–Wallis rank sum test 32, 33), and the mean effect of correction was significant via one‐way ANOVA (which perhaps erroneously assumes normality; *P* < 0.05 in both cases). We believe that the effect of the method to increase overlap between odd‐ and even‐echoes effectively is indicative of its utility in correcting distortions, as no differences in signal intensity between summed odd and even echoes could be visually observed in the data. All data presented are provided as examples in the source code repository for the method, and all statistical comparisons were performed in R 34.

## DISCUSSION AND CONCLUSION

We have shown that existing image‐domain distortion correction algorithms can be applied with slight modification to hyperpolarized EPI datasets that necessarily do not have consistently high SNR within a small, singly‐connected region of the acquired image. The method we have proposed requires no additional reference scan, and has a spatial region of support that is determined entirely from the acquired data. As hyperpolarized compounds may be present with evolving shifts in regions that are spatially distinct from structures that could conceviably be localized via proton references the use of reference‐free methods is attractive. For example, while hyperpolarized pyruvate has been used to characterize the inflammatory response of the lung to injury 35, 36, 37, it is likely to not be possible to obtain reference proton *B*_0_ maps of the lung parenchyma with the same spatial extent as the acquired hyperpolarized data owing to the low tissue density within the lung.

In regions far away from the heart, such as the liver and abdominal aorta, the method was able to correct for oscillating spatial shifts that could not be corrected for via bulk frequency adjustments (cf. inferior slices in Supporting Videos S1–2 and S5–6). As it is not possible to ensure *B*_0_ homogeneity across the entire volume of a subject, it is usually the case that the shimming process optimizes *B*_0_ homogeneity over a region of interest, chosen here to be the heart. Typically the *B*_0_ homogeneity in regions outside the region of interest therefore decreases as a consequence of the design of spherical harmonic shimsets. The use of image‐domain distortion correction algorithms therefore allows for the amelioration of artefacts in regions that may be of secondary interest when compared to the primary region of interest that was initially proscribed for the experiment, although we stress that the method assumes that inherent chemical shift differences are already accounted for prior to its invocation. Similarly, as it is not readily possible to rapidly repeat hyperpolarized experiments should an error be discovered, such techniques therefore minimize the cost of a slight error in FOV planning.

Like all image domain methods, that proposed here relies upon sufficient SNR in the acquired data. In this work, the use of regularization and progressive subsampling limits the potentially deleterious effects of noise by effectively initially low‐pass filtering the acquired data to ensure that smoothly varying *B*_0_ fluctuations are corrected for first, and that the returned deformation mesh is smoothly varying. In the limit as SNR tends to zero, it is therefore expected to be relatively robust, and return **b** that correspond to a generalized identity matrix. This property is advantageous in hyperpolarized experiments, where the period of acquisition is usually chosen to contain a set of zero SNR images either side of the bolus of injected contrast agent.

It is difficult to directly compare distortion correction methods for hyperpolarized datasets, as there is no underlying “ground truth” image with which they can be compared. Likewise, information theory metrics such as image self‐similarity or mutual information that measure generalized similarity between images by statistical means do not provide a useful metric for comparison between multidimensional images with drastically different intensity profiles, such as that examined here. The quantitative comparison undertaken here is simplistic, and relies upon thresholding the images into “signal” and “noise,” a process that is potentially subjective and inherently neglects intensity variation. Consequently, a quantitative framework to assess hyperpolarized distortion correction methods represents an area for future work.

The use of image‐domain distortion correction algorithms, such as that proposed here, can ameliorate susceptibility artefacts present in all EPI readouts, including those of hyperpolarized experiments. The nonuniform and temporally varying SNR necessarily present within hyperpolarized experiments necessitates care in their application to the data. We have shown a particular algorithm is able to ameliorate image‐domain shifts and better coregister acquired data to underlying anatomy.

## Supporting information

Supporting Information Figure 1.

Supporting Information Figure 2.

Supporting Information Figure 3.

Supporting Information Figure 4.

Supporting Information Figure 5.

Supporting Information Figure 6.

**Videos S1–6**: Original (odd‐numbered) and distortion‐corrected (even‐numbered) example datasets. For each video, three rows of images are shown corresponding to hyperpolarized [1‐^13^C]pyruvate, ^13^C‐bicarbonate and [1‐^13^C]lactate respectively, and the z axis is shown concatenated across rows, with the head to the left. The TR for each metabolite is approximately 1.2−1.4 s, which is not depicted here.

**Videos S7–8**: Original 7 and distortion‐corrected 8 single‐slice data overlaid on an anatomical proton reference. Columns refer to pyruvate, bicarbonate and lactate images respectively. The colour axis is scaled relative to the maximum intensity in the image with values < 0.2 set to transparent.
