# Supplementary figures and images for: Susceptibility‐induced distortion correction in hyperpolarized echo planar imaging
Source: Magn Reson Med. 2017 Jul 19;79(4):2135–41. doi: 10.1002/mrm.26839 (PMC5836862; doi:10.1002/mrm.26839)

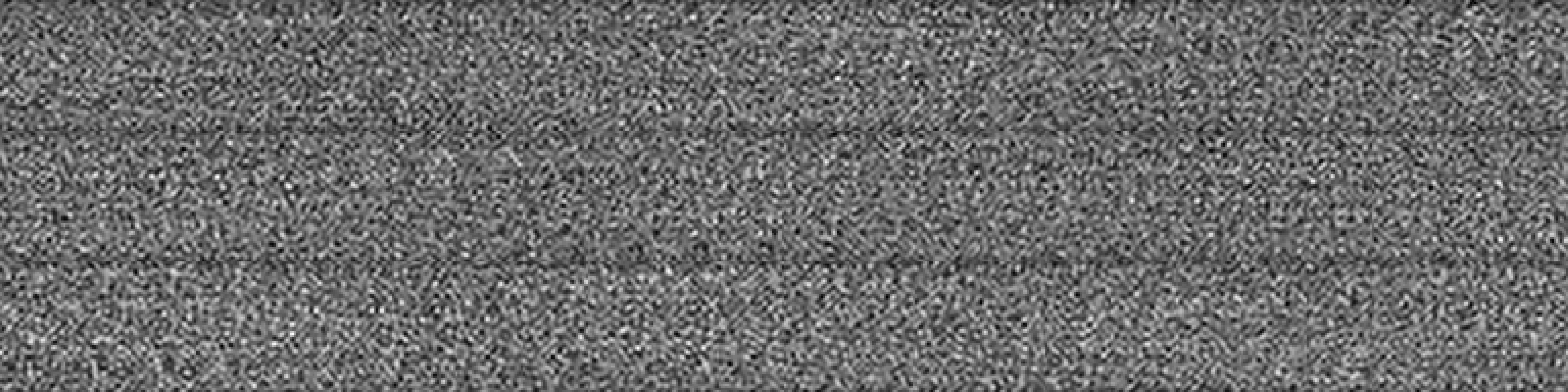

Supplement: Supplementary file 1 — Supporting Information Figure 1. [file MRM-79-2135-s001.gif]

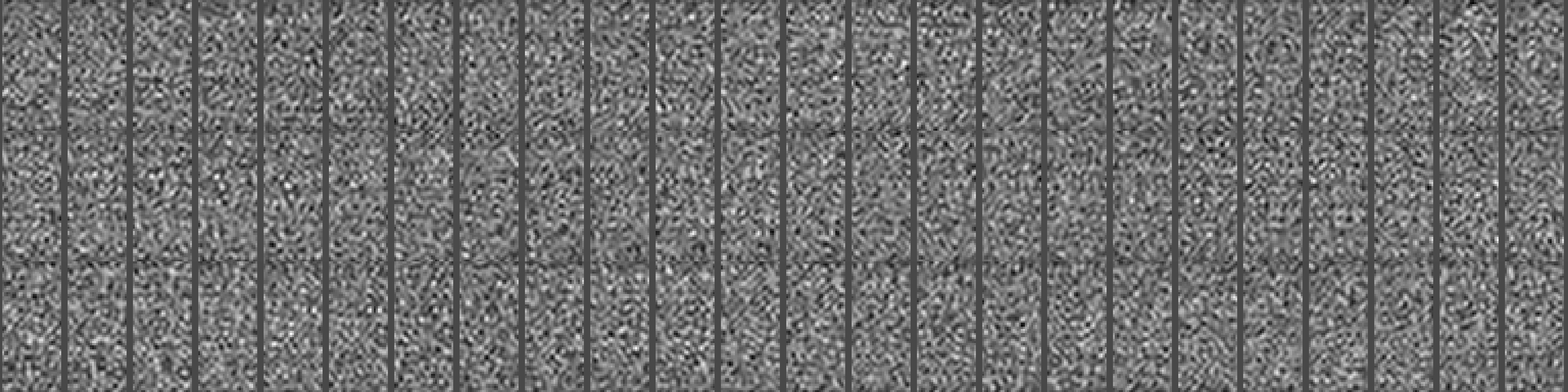

Supplement: Supplementary file 2 — Supporting Information Figure 2. [file MRM-79-2135-s002.gif]

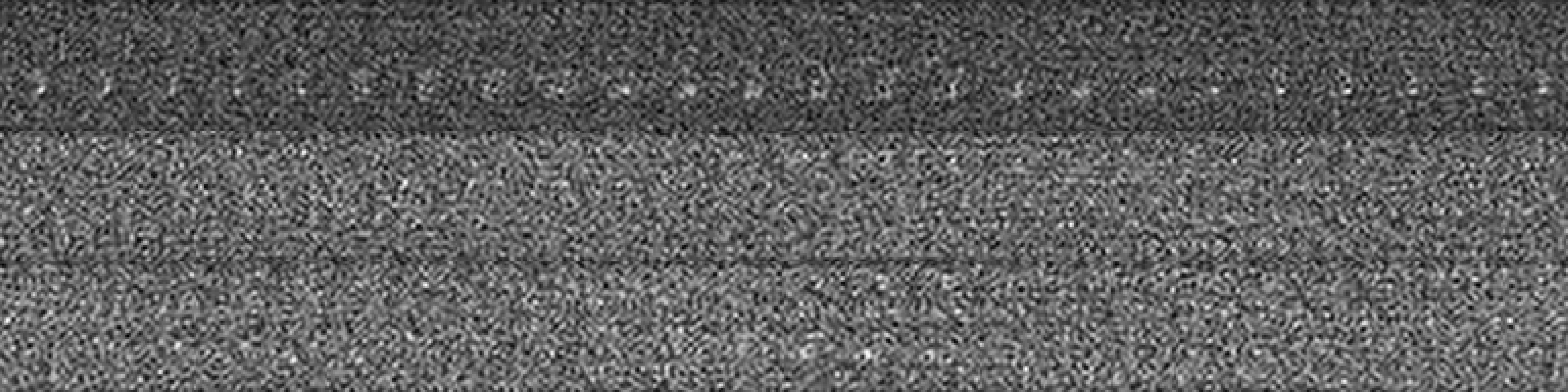

Supplement: Supplementary file 3 — Supporting Information Figure 3. [file MRM-79-2135-s003.gif]

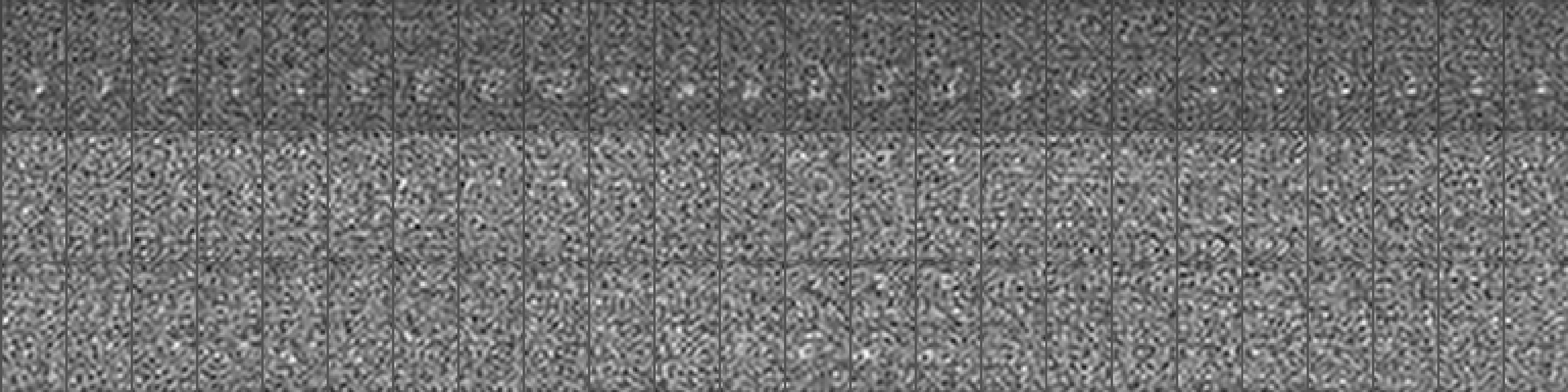

Supplement: Supplementary file 4 — Supporting Information Figure 4. [file MRM-79-2135-s004.gif]

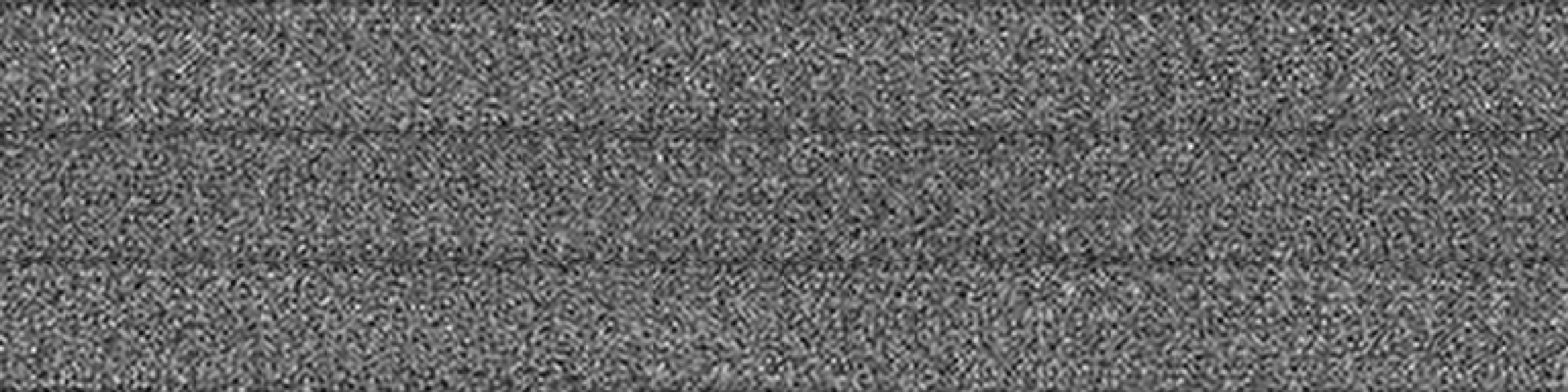

Supplement: Supplementary file 5 — Supporting Information Figure 5. [file MRM-79-2135-s005.gif]

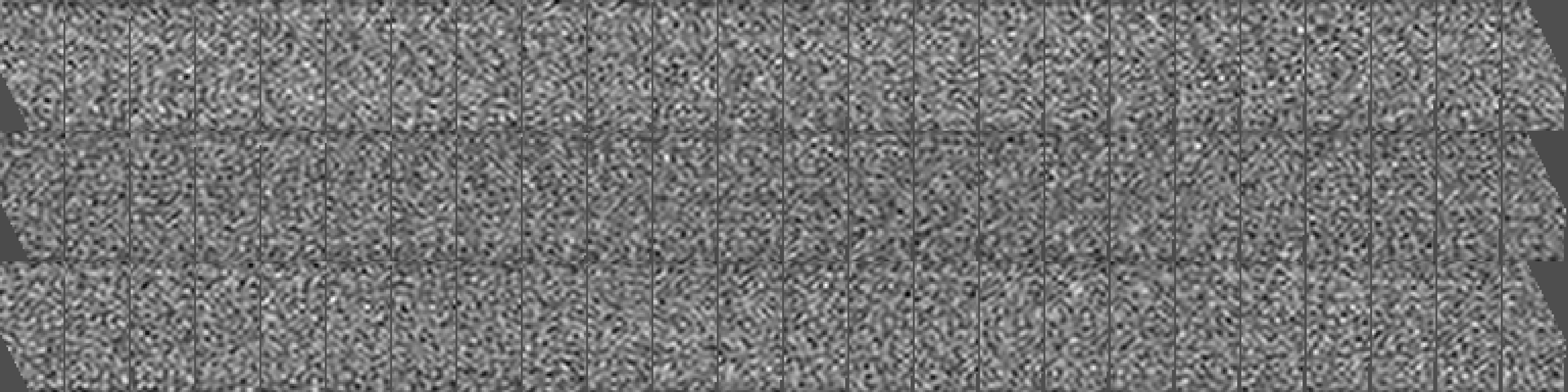

Supplement: Supplementary file 6 — Supporting Information Figure 6. [file MRM-79-2135-s006.gif]
